# Supplementary material for: Ensemble Generalization of the Perdew–Zunger Self-Interaction Correction: A Way Out of Multiple Minima and Symmetry Breaking
Source: J Chem Theory Comput. 2024 Aug 14;20(16):7144–54. doi: 10.1021/acs.jctc.4c00694 (PMC11360130; doi:10.1021/acs.jctc.4c00694)
Supplement: Supplementary file 1 — ct4c00694_si_001.pdf [file ct4c00694_si_001.pdf]

Supporting Information: Ensemble Generalization of the Perdew–Zunger Self-Interaction Correction: a Way Out of Multiple Minima and Symmetry Breaking

Sebastian Schwalbe,[*,†,‡,¶] Wanja Timm Schulze,[†,§] Kai Trepte,[‖] and Susi Lehtola[*,⊥]

†*Contributed equally to this work*

‡*Center for Advanced Systems Understanding (CASUS), D-02826 Görlitz, Germany*

¶*Helmholtz-Zentrum Dresden-Rossendorf (HZDR), D-01328 Dresden, Germany*

§*Institute for Physical Chemistry, Friedrich Schiller University, D-07743 Jena, Germany*

‖*Taiwan Semiconductor Manufacturing Company North America, San Jose, USA*

⊥*Department of Chemistry, University of Helsinki, P.O. Box 55, FI-00014 Helsinki, Finland*

E-mail: s.schwalbe@hzdr.de; susi.lehtola@alumni.helsinki.fi

Optimized ensemble FOD configurations of benzene (C_6H_6) are shown in fig. S1 for calculations with the SPW92 LDA functional. Although the structures in figs. S1a and S1b appear at first glance strikingly different, it is important to realize that the ensemble calculation employs a superposition of both structures. It is easy to see that the superposition of LT1 and LT2 in fig. S1a can be visualized as in fig. S1b by using different colors to illustrate the FODs of different members of the ensemble. Moreover, the α and β FODs of LDQ1 and

LDQ2 are related by interchange: $\alpha \leftrightarrow \beta$ coincides with LDQ1 $\leftrightarrow$ LDQ2.

Results of the calculations with the PBE GGA and TPSS meta-GGA functionals are shown in table S1 and fig. S2 and in table S2 and fig. S3, respectively. Like the SPW92 data in the main text, these data demonstrate that FLO-SIC calculations based on LT FODs predict a symmetry-broken molecular geometry, while FLO-SIC calculations based on LDQ FODs break spin symmetry, as we have previously shown in ref. S1. In contrast, the E-FLO-SIC method of this work eliminates all such symmetry breaking, predicting a symmetric molecular geometry without spin polarization.

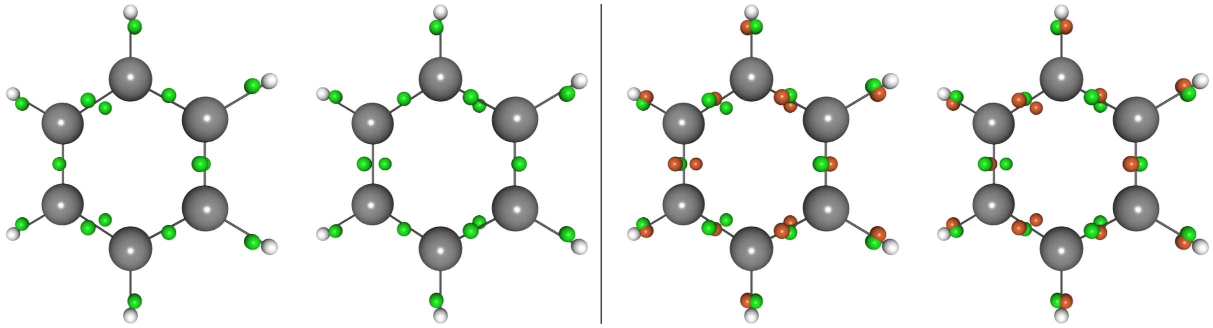

(a) {LT1,LT2}

(b) {LQD1,LDQ2}

Figure S1: Optimized ensemble FOD configurations of benzene using the SPW92 LDA functional, visualized with PyFLOSIC2:GUI.[S2,S3] Carbon atoms are colored in grey, hydrogens in white, and the FODs in green and red denoting spin-up and spin-down FODs. In case the FOD positions in α and β spin channels are identical, only the green FODs are visualized.

Table S1: Properties of the optimum geometry of benzene and the corresponding wave function in FLO-SIC and E-FLO-SIC calculations with PyFLOSIC2 using the PBE GGA functional. The optimal bond lengths of short and long bonds $d_{\mathrm{CC}}^{\mathrm{short}}$ and $d_{\mathrm{CC}}^{\mathrm{long}}$ coincide in the case of a symmetric optimum geometry, but differ for a symmetry-broken optimal geometry. The value of the KS total energy E^{KS} is minimized in the DFT calculation, but competes with the SIC in (E-)FLO-SIC calculations that minimize the total energy $E^{\mathrm{(E\text{-})FLO\text{-}SIC}}$, instead. For reference, the $\langle \hat{\boldsymbol{S}}^2 \rangle$ is also shown.

	$d_{\mathrm{CC}}^{\mathrm{short}}$ [Å]	$d_{\mathrm{CC}}^{\mathrm{long}}$ [Å]	E^{KS} [E_{h}]	$E^{\mathrm{(E\text{-})FLO\text{-}SIC}}$ [E_{h}]	$\langle \hat{\boldsymbol{S}}^2 \rangle$
DFT	1.392	1.392	−231.899976	-	0.000
Configuration/Ensemble					
LT1/LT2	1.342	1.417	−231.861310	−231.882215	0.000
LDQ1/LDQ2	1.392	1.392	−231.864792	−231.877081	0.157
Ensemble	1.392	1.392	−231.880753	−231.856230	0.000

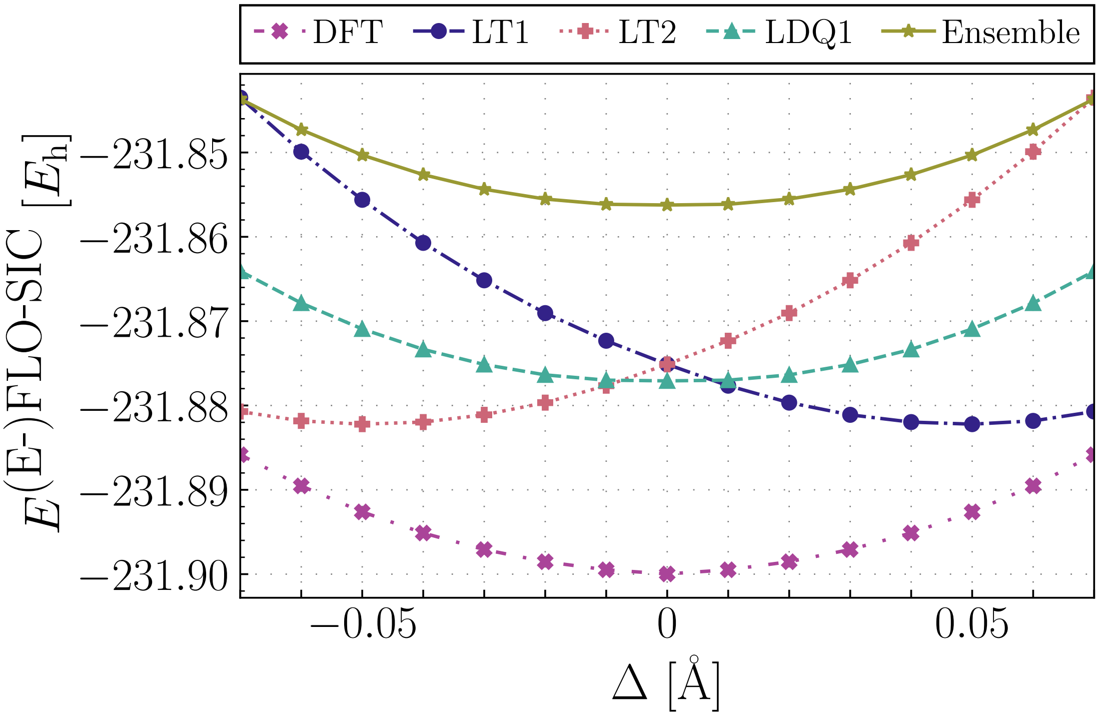

Figure S2: FLO-SIC and E-FLO-SIC total energies as a function of the local distortion Δ using the PBE GGA functional, visualized with Matplotlib.[S4] Data for the LDQ2 calculation is not shown, because it is indistinguishable from the shown LDQ1 data. As the {LT1,LT2} and {LDQ1,LDQ2} calculations were initialized from equivalent fodMC starting points (see main text), their results are indistinguishable and have been marked here as “ensemble”. In-depth details on the minima of each calculation are given in table S1.

Table S2: Properties of the optimum geometry of benzene and the corresponding wave function in FLO-SIC and E-FLO-SIC calculations with PyFLOSIC2 using the TPSS meta-GGA functional. The optimal bond lengths of short and long bonds d_{CC}^{short} and d_{CC}^{long} coincide in the case of a symmetric optimum geometry, but differ for a symmetry-broken optimal geometry. The value of the KS total energy E^{KS} is minimized in the DFT calculation, but competes with the SIC in (E-)FLO-SIC calculations that minimize the total energy $E^{(E-)FLO-SIC}$, instead. For reference, the $\langle \hat{\boldsymbol{S}}^2 \rangle$ is also shown.

	d_{CC}^{short} [Å]	d_{CC}^{long} [Å]	E^{KS} [E_h]	$E^{(E-)FLO-SIC}$ [E_h]	$\langle \hat{\boldsymbol{S}}^2 \rangle$
DFT	1.392	1.392	−232.252025	-	0.000
Configuration/Ensemble					
LT1/LT2	1.352	1.412	−232.225290	−231.790291	0.000
LDQ1/LDQ2	1.392	1.392	−232.227232	−231.787078	0.119
Ensemble	1.392	1.392	−232.238698	−231.771945	0.000

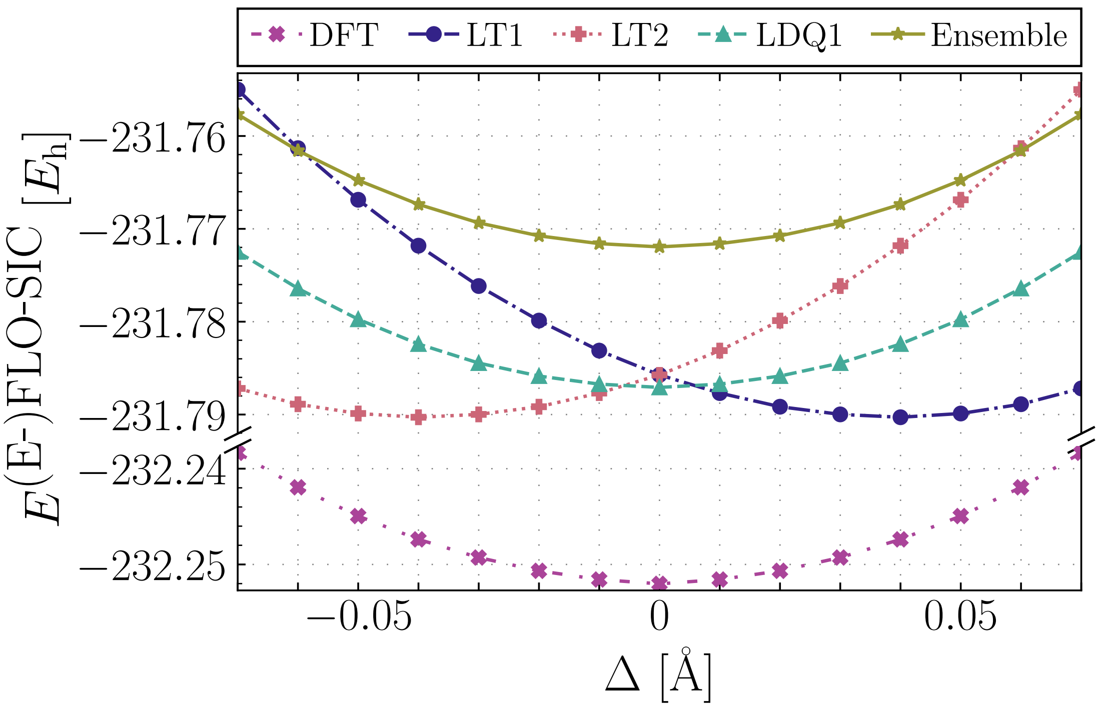

Figure S3: FLO-SIC and E-FLO-SIC total energies as a function of the local distortion Δ using the TPSS meta-GGA functional, visualized with Matplotlib.[S4] Data for the LDQ2 calculation is not shown, because it is indistinguishable from the shown LDQ1 data. As the {LT1,LT2} and {LDQ1,LDQ2} calculations were initialized from equivalent fodMC starting points (see main text), their results are indistinguishable and have been marked here as "ensemble". In-depth details on the minima of each calculation are given in table S2.

References

(S1) Trepte, K.; Schwalbe, S.; Liebing, S.; Schulze, W. T.; Kortus, J.; Myneni, H.; Ivanov, A. V.; Lehtola, S. Chemical bonding theories as guides for self-interaction corrected solutions: Multiple local minima and symmetry breaking. *J. Chem. Phys.* **2021**, *155*, 224109.

(S2) Schwalbe, S.; Fiedler, L.; Kraus, J.; Kortus, J.; Trepte, K.; Lehtola, S. PyFLOSIC: Python-based Fermi–Löwdin orbital self-interaction correction. *J. Chem. Phys.* **2020**, *153*, 084104.

(S3) Liebing, S.; Trepte, K.; Schwalbe, S. *Springer Proceedings in Physics*; Springer International Publishing, 2022; pp 167–186.

(S4) Hunter, J. D. Matplotlib: A 2D Graphics Environment. *Comput. Sci. Eng.* **2007**, *9*, 90–95.
